# Bibliometric and trend analysis of the top 100 most-cited articles on anterior lumbar interbody fusion (ALIF)

**DOI:** 10.1016/j.bas.2026.106042

**Published:** 2026-04-10

**Authors:** Badr Hafiz, Thamer Alsharif, Abdulaziz Hamzah, Faisal Sukkar, Yousef Bassi, Moaath Alghamdi, Muhammad Tariq, Rakan Bokhari, Saleh Baeesa

**Affiliations:** a Department of Neurosciences, King Faisal Specialist Hospital and Research Centre, Jeddah, Saudi Arabia; b Neurosurgery Department, King Abdulaziz Specialist Hospital, Taif, Saudi Arabia; c Neurosurgery Section, Department of Surgery, King Abdulaziz University Hospital, Jeddah, Saudi Arabia; d College of Medicine, King Abdulaziz University, Jeddah, Saudi Arabia; e Faculty of Medicine, Alfaisal University, Riyadh, Saudi Arabia

**Keywords:** Bibliometric analysis, Anterior lumbar interbody fusion (ALIF), Citation classics, Collaborative networks, Intellectual structure, Spine surgery

## Abstract

**Introduction:**

Anterior lumbar interbody fusion (ALIF) is a widely utilized spinal procedure supported by an expanding body of literature. However, the structure, influence, and thematic evolution of the most impactful studies remain incompletely characterized. This study aimed to analyze the bibliometric profile of the 100 most-cited ALIF publications.

**Methods:**

A bibliometric analysis was performed using the Web of Science Core Collection. The 100 most-cited ALIF-related articles were identified from database inception through 2025. Extracted data included authorship, journals, institutions, countries, and citation metrics. Analyses used the Bibliometrix R package to evaluate productivity, collaboration, and keyword trends.

**Results:**

Dataset included 463 authors across 28 journals, with a mean of 5.4 authors per article and international collaboration (9%). The literature demonstrated high impact, with a mean document age of 15.2 years and an average of 125.4 citations per article. Publication output was concentrated in *Spine* (19%) and *The Spine Journal* (17%), while the United States contributed the highest citation share. The most-cited studies reached 365 citations, whereas recent publications showed high citation velocity (up to 32.4 citations/year). Research focus evolved from fusion outcomes and biomechanics toward complications and minimally invasive approaches. Most studies were retrospective.

**Conclusion:**

Influential ALIF literature is highly concentrated and shaped by both historically cited studies and emerging high-impact work. The findings highlight a potential divergence between citation prominence and evidence hierarchy, emphasizing the need for critical appraisal and more robust prospective research. This analysis provides a structured framework to understand historical trends, current priorities, and future directions in ALIF research.

## Highlights

• First bibliometric analysis of Top-100 most-cited ALIF publications.
• Dataset included 463 authors across 28 journals, showing high collaboration.
• *Spine* and *The Spine Journal* were the dominant publication sources.
• Citation trends showed both landmark work and high-velocity recent studies.
• USA led global citation influence, followed by Korea and Australia.

## 1 Introduction

Since its first description in the early 20th century, including early reports such as Burns in 1933 (Burns, 1933), anterior lumbar interbody fusion (ALIF) has evolved into a widely accepted surgical option for a broad range of lumbar pathologies, from degenerative spondylolisthesis to complex sagittal plane deformities (Burns, 1933; Hsieh et al., 2007; Tu et al., 2021; Rao et al., 2015). However, these early descriptions predate modern indexing systems and differ substantially from contemporary surgical techniques. The evolution of ALIF into its modern form occurred in the late 20th century with the introduction of standardized surgical approaches and implant technologies. Accordingly, the present analysis reflects the modern citation era of ALIF, as captured within indexed bibliographic databases.

The primary clinical advantage afforded by ALIF, namely the superior and reliable restoration of disc height and segmental lordosis through its large-footprint interbody spacer, remains unmatched by posterior approaches (Mobbs et al., 2015; Phan et al., 2015). By utilizing the retroperitoneal corridor, surgeons can avoid iatrogenic trauma to the paraspinal musculature and neural structures, accelerating postoperative recovery and reducing long-term morbidity (Teng et al., 2017; Mummaneni et al., 2021).

However, the technical complexity of ALIF, particularly the requirement for meticulous vascular mobilization, has restricted adoption by members of the surgical community (Inamasu and Guiot, 2006; Brau, 2002). The integration of “stand-alone” interbody cages, introduction of osteobiologics such as recombinant human bone morphogenetic protein-2 (rhBMP-2), and recent adoption of robotic-assisted navigation have all sought to refine the safety and efficacy of the procedure (Burkus et al., 2005; Sasso et al., 2003). These innovations have sparked exponential growth in the peer-reviewed literature, creating a vast and often fragmented evidence base (Chen et al., 2018; Li et al., 2025). For contemporary spine surgeons, the challenge is no longer a lack of data but rather the difficulty in identifying the relevant works contributing to fundamentally altered clinical paradigms (Hu et al., 2024; de Kunder et al., 2018).

Unlike meta-analyses, which address predefined clinical questions, bibliometric analysis identifies the intellectual structure, historical inflection points, and influential evidence that shape clinical decision-making. It offers a rigorous, quantitative method for navigating this information density (van Eck and Waltman, 2010). Unlike traditional systematic reviews, bibliometrics maps the intellectual landscape of a field by identifying the most influential publications, tracking temporal shifts in research priorities, and characterizing patterns of knowledge production, authorship networks, and geographic contributions (Aria and Cuccurullo, 2017; Lin et al., 2022). By analyzing the most-cited studies, it becomes possible to trace the progression of ALIF research from early anatomical and technical descriptions to contemporary clinical trials, technological innovations, and outcome-driven investigations (Kuslich et al., 1998; Bagby, 1988). Furthermore, this approach highlights the key journals, institutions, and regions that have shaped the direction of anterior spinal surgery over time (Stauffer and Coventry, 1972; Mobbs et al., 2013). In the context of ALIF, where the literature spans decades of evolving techniques, implants, and indications, bibliometric analysis provides a complementary perspective that contextualizes outcome-based evidence within the broader intellectual and historical development of the field.

Despite the bibliometric prominence of ALIF, a comprehensive assessment of its most influential literature has not yet been conducted. Identifying the papers that have achieved “citation classic” status is essential for understanding the evidence-based origins of current practice and guidelines as well as for identifying persistent gaps in knowledge (Lightsey et al., 2022; Issa et al., 2025). This study aimed to perform a detailed bibliometric mapping of the 100 most-cited ALIF articles within the Web of Science Core Collection, providing a roadmap of where the field has been and where future innovations are likely to lead.

## 2 Methodology

### 2.1 Study design

This study presents a bibliometric analysis of the most influential literature on ALIF. We defined this as the 100 highest-cited publications in a single indexed database. The analysis was conducted in accordance with the predefined study protocol outlined in the protocol, with the objective of characterizing the publication patterns, citation performance, and collaborative structures within the high-impact ALIF literature.

### 2.2 Data source

The Web of Science Core Collection, specifically the Science Citation Index Expanded, was used as the bibliographic data source for this study. Restricting analysis to a single, well-established database minimizes the citation metric heterogeneity and facilitates a reproducible bibliometric assessment.

### 2.3 Search strategy and study selection

A structured topic search was conducted using ALIF-relevant terms centered on “anterior lumbar interbody fusion” and “ALIF,” as specified in the protocol. Retrieved records were filtered to include English-language publications only and were limited to Articles and Reviews to ensure the inclusion of peer-reviewed, citable scholarly outputs. The search was performed from database inception. Following retrieval, records were ranked according to “Times Cited,” as defined by the Web of Science at the time of data export. The top 100 most-cited records were retained for analysis. This selection strategy was intentionally aligned with the study objective of characterizing the most influential ALIF literature rather than the entirety of ALIF-related publications. Records not primarily focused on ALIF, including those centered on other spinal approaches or regions, as well as non-article formats such as editorials or letters, were excluded in accordance with the predefined eligibility framework. The earliest ALIF-related publication identified dated back to 1961, but the earliest included study was published in 1988, corresponding to the early phase of modern ALIF practice with the introduction of contemporary techniques and instrumentation.

### 2.4 Data extraction

For each eligible publication, bibliographic metadata and bibliometric indicators were extracted. The extracted variables included publication year, source journal, authorship, and institutional affiliation data. Citation performance indicators comprised total citations, citations per year, and normalized citation measures, where available in bibliometrix outputs. All extracted data were handled in accordance with the predefined analytic plan and were not supplemented with external citation sources.

### 2.5 Bibliometric analysis and visualization

Bibliometric analyses were performed using the Bibliometrix package in R, supplemented by visualization workflows consistent with the proposal. Productivity and concentration analyses were conducted at multiple levels, including sources (most relevant journals and source-level H-index within the retrieved set), authors (most relevant authors by full counting and fractionalized contribution, as well as author impact metrics), affiliations (most represented institutions), and countries (scientific production and citation prominence). Bradford's law visualization was applied to evaluate the degree of journal concentration within the top-cited ALIF literature and to identify core sources. Collaboration structures were examined using network visualizations of co-authorship and country-level collaboration, while thematic signals were summarized through keyword-based outputs. All analyses were descriptive in nature, and all reported findings were derived exclusively from the uploaded figures and tables without extrapolation beyond the available data.

## 3 Results

### 3.1 Dataset profile and core bibliometric descriptors

The final analytic dataset comprised 100 highly cited ALIF publications published between 1988 and 2025. These papers were distributed across 28 journals, indicating that influential ALIF research is concentrated in a limited number of sources rather than in a single outlet. Authorship was strongly collaborative in nature. A total of 463 unique authors contributed to the dataset, whereas only two documents were single-authored. The mean collaboration intensity was 5.4 authors per document, and 9% of the papers involved international co-authorship. The dataset included 312 author keywords, providing a solid foundation for thematic analysis. At the document level, the mean document age was 15.2 years, and the average citation count was 125.4 citations per paper, confirming that the dataset represents a mature, highly influential body of ALIF literature rather than predominantly recent publications.

### 3.2 Temporal distribution of scientific production

**Fig. 1:**
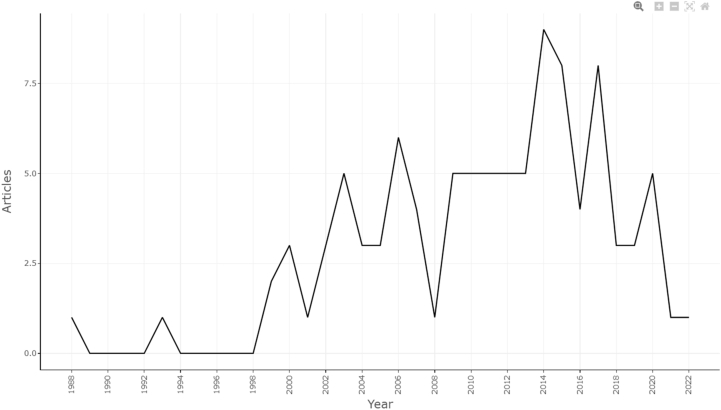
Line plot depicting the annual scientific production of top-cited ALIF papers (1988–2022).

The annual scientific production of ALIF-related literature demonstrated a fluctuating pattern over time (Fig. 1). In the early years, output was minimal, with only one article published in 1988 and no publications recorded across several subsequent years in the early 1990s. Scientific production began to increase gradually from the late 1990s, reaching 2–3 articles annually between 1999 and 2002, and rising further to 5–6 articles per year in the mid-2000s. A more pronounced expansion was observed in the following decade, with consistent outputs of approximately five articles per year between 2009 and 2013. The peak in annual production occurred in 2014 (nine articles) and remained relatively high in 2015 and 2017 (eight articles each). In recent years, output declined again, with three to five articles published between 2018 and 2020 and only one published annually in 2021 and 2022. Overall, rather than a smooth growth trajectory, ALIF scientific production occurred in intermittent clusters, reflecting periods of intensified research activity, followed by a relative decline. This temporal distribution indicates the presence of both early foundational contributions and later phases of increased attention in the field. This pattern of fluctuating scientific production resembles previously described models of research activity, such as “Scott's parabola,” where periods of accelerated growth are followed by phases of stabilization or decline in publication output.

### 3.3 Citation dynamics over time

**Fig. 2:**
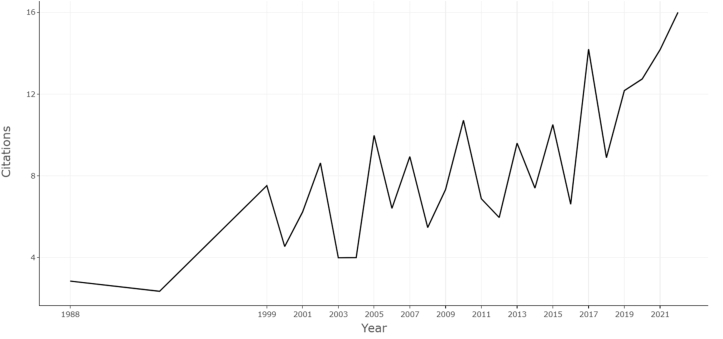
Line plot illustrating the average citations per year for the top-cited ALIF publications.

The average citations per year increased in the later part of the timeline (Fig. 2). Although year-to-year variability was evident, the overall trend shows higher citation activity in recent years. This reflects the continued influence of established ALIF studies and the emergence of newer publications with high citation velocity, consistent with the TC/year values observed for globally cited documents.

### 3.4 Journal distribution and source impact

**Fig. 3:**
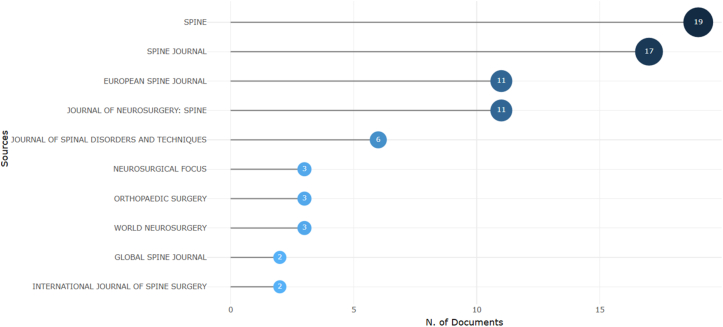
Horizontal bubble chart of the most relevant journals contributing to top-cited ALIF literature.

**Fig. 4:**
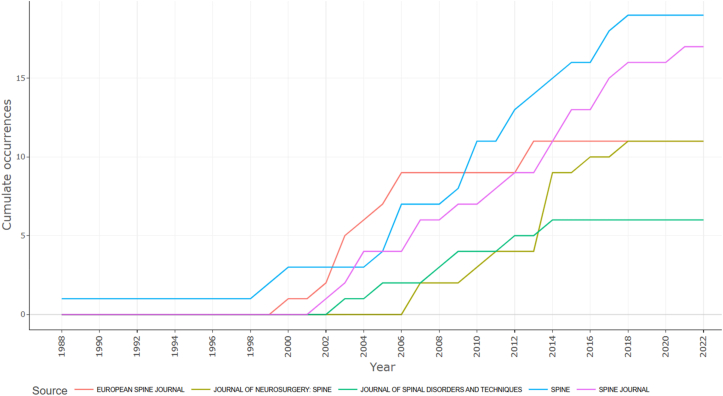
Cumulative line chart depicting the source impact over time for leading ALIF journals.

**Fig. 5:**
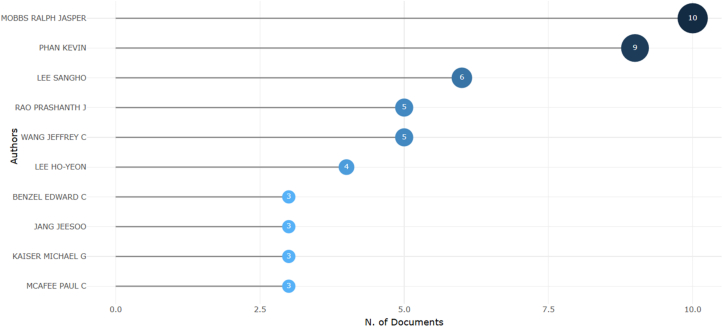
Horizontal bubble chart of the most productive authors in the top-cited ALIF corpus (full count).

Journal productivity in the top-cited ALIF literature was highly concentrated (Fig. 3). *Spine* contributed the largest number of papers (19 documents), followed closely by *The Spine Journal* (17 documents). The second tier included *the European Spine Journal* and *Journal of Neurosurgery: Spine*, each contributing 11 papers. Beyond these core journals, contributions declined sharply, with fewer distributed across several additional outlets. The source H-index within the top-cited dataset closely mirrored this pattern, with *Spine* (H = 19) and *The Spine Journal* (H = 17) leading these rankings. The evolution of source impact over time (Fig. 4) shows stepwise growth rather than steady annual increases, indicating that highly cited contributions from leading journals have accumulated across multiple publication years. This concentration pattern is further supported by Bradford's Law, which identifies a small core of journals responsible for a disproportionately large share of influential ALIF publications (Fig. 5).

### 3.5 Author productivity and impact

Author-level analysis showed that contributions to the top-cited ALIF literature were concentrated among a limited group of authors (Fig. 5). Ralph Jasper Mobbs was the most prolific author with 10 publications, followed by Kevin Phan (9 publications) and Sangho Lee (6 publications). When fractional authorship was considered, the same authors remained dominant, although their fractionalized counts were lower than their full counts, reflecting the multi-author nature of ALIF research. Mobbs (2.77 fractionalized articles) and Phan (2.39) were the leading contributors.

**Fig. 6:**
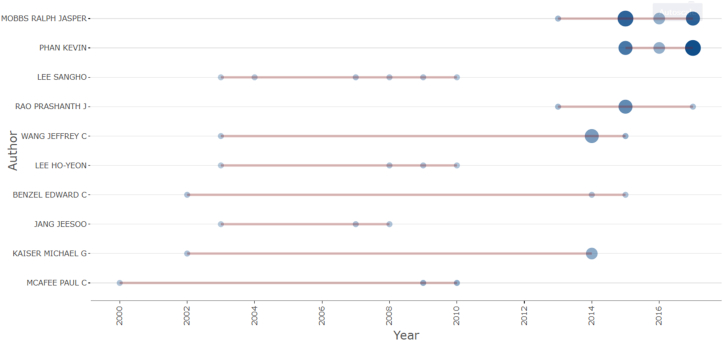
Timeline bubble chart of the authors production over time for the leading ALIF contributors.

Author production over time (Fig. 6) demonstrates that leading authors contributed during specific periods rather than evenly across the entire timeline, with many showing activity concentrated in later years. Author impact, measured using the H-index within the top-cited dataset, followed a similar pattern, with Mobbs (H = 10) and Phan (H = 9) ranking the highest.

### 3.6 Institutional contributions

**Fig. 7:**
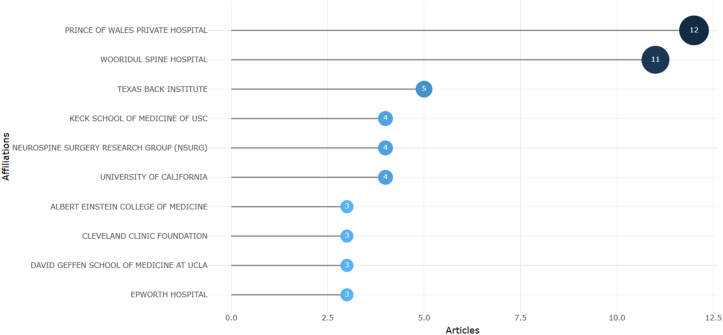
Horizontal bubble chart of the most frequently represented institutional affiliations.

Institutional analysis revealed repeated representations from a small number of affiliations (Fig. 7). Prince of Wales Private Hospital and Woodridge Spine Hospital were the most frequently occurring institutions, followed by several others that appeared multiple times in the dataset. This clustering suggests that high-impact ALIF research is concentrated in a limited number of specialized centers.

### 3.7 Most globally cited ALIF publications

**Fig. 8:**
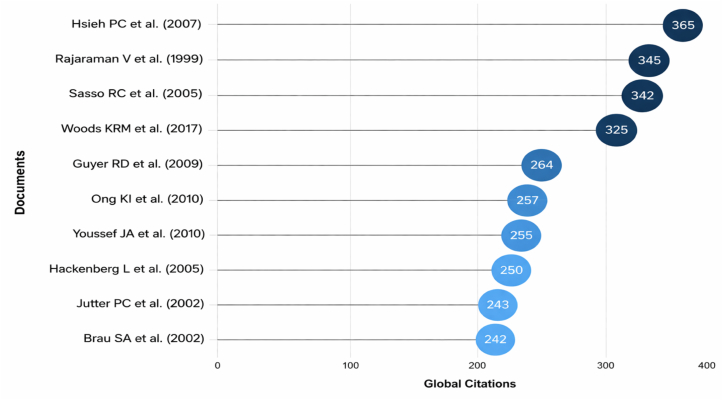
Horizontal bubble chart of the most globally cited ALIF publications and their citation indicators.

The most globally cited documents exhibited substantial variations in both total citation counts and citation velocity (Fig. 8). The most cited paper was Hsieh et al. (2007) with 356 citations, followed closely by Rajaraman et al. (1999) and Sasso et al. (2005), each exceeding 341 citations. Notably, some newer papers demonstrated exceptionally high citation rates, such as Woods et al. (2017), which showed the highest TC/yr value. Across the broader top-100 cited papers, total citations ranged from 365 to 71, indicating a wide spread even within the elite citation tier (Table 1). Together, these findings show that the ALIF literature includes both older citation-rich studies and newer high-velocity publications, with normalized citation metrics providing additional insights beyond raw totals.

**Table 1:** Summary of predefined bibliometric study protocol.

Component	Specification
Study design	Descriptive bibliometric analysis
Objective	To characterize publication patterns, citation impact, and collaboration in high-impact ALIF literature
Database	Web of Science Core Collection (SCI-Expanded)
Search terms	“anterior lumbar interbody fusion” OR “ALIF”
Eligibility	English-language Articles and Reviews focused on ALIF
Ranking method	Web of Science “Times Cited”
Sample	Top 100 most-cited publications
Data extracted	Publication year, journal, authors, affiliations, citations
Analysis tools	bibliometrix package (R)

### 3.8 Keyword landscape and dominant terms

The analysis of author keywords provides a descriptive overview of the most prominent terms identified in the bibliometric workflow. The most frequent terms were the core procedural descriptors “anterior,” “interbody,” “fusion,” and “lumbar,” confirming that the retrieved corpus is topically anchored to ALIF in accordance with the study selection criteria. Additional recurrent terms included comparative framing language such as “versus” and “comparison,” as well as clinically oriented terms such as “outcomes,” “complications,” and broader spinal descriptors including “spine.” Overall, the keyword distribution reflects a strong focus on procedural characterization and comparative clinical evaluation within the top-cited ALIF literature without implying a predefined thematic clustering.

### 3.9 Collaboration patterns and country-level contributions

**Fig. 9:**
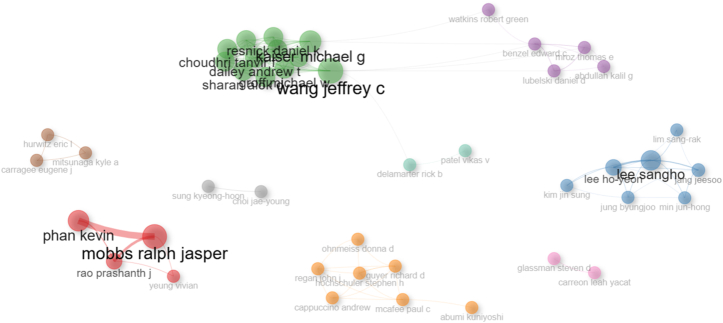
Author collaboration network map in top-cited ALIF literature.

The author collaboration network illustrates multiple distinct collaboration clusters rather than a single, unified network (Fig. 9). Dense connections within clusters and fewer links between clusters suggest the presence of separate research communities, consistent with the moderate level of international collaboration observed in this dataset.

**Fig. 10:**
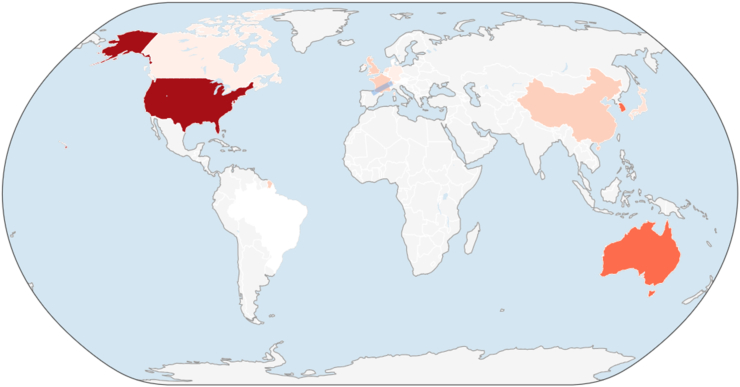
Choropleth map depicting country-level citation distribution of top-cited ALIF publications.

At the country level, the United States was the dominant contributor, accounting for 6489 citations, far exceeding that of all other countries (Fig. 10). Korea and Australia formed a secondary tier, followed by a broader group of contributing countries with substantially lower citation totals. This distribution demonstrates a steep citation gradient, with the United States leading the global influence in highly cited ALIF research.

## 4 Discussion

From a historical perspective, the evolution of ALIF is closely reflected in the citation patterns identified in this study. Early descriptions of anterior lumbar fusion date back to the early 20th century, with subsequent refinement in the late 1980s marking the beginning of the modern ALIF era, particularly with the introduction of improved instrumentation and interbody devices (Burns, 1933; Bagby, 1988; Stauffer and Coventry, 1972). The earliest highly cited studies in our dataset predominantly focus on fusion techniques, grafting strategies, and biomechanical stability (Kuslich et al., 1998; Loguidice et al., 1988; Dennis et al., 1989). Over time, this focus shifted toward complication profiles, including vascular injury and retrograde ejaculation (Baker et al., 1993; Brau et al., 2004; Sasso et al., 2003), followed by more recent emphasis on implant technology, osteobiologics, and minimally invasive approaches (Burkus et al., 2005; Guyer et al., 2009). This progression highlights how citation trends parallel the broader surgical evolution of ALIF from foundational technique development to optimization of safety and outcomes.

This bibliometric analysis characterizes the intellectual structure and thematic evolution of the highly cited literature on ALIF and closely related anterior/lateral interbody strategies. Interpreting the results at the level of citation influence is valuable because it reflects enduring clinical problems, technical inflection points, and evidence consolidation within specialty journals and collaborative author groups. Importantly, analysis of the highly cited publications indicates that the literature most frequently referenced by the field focuses on surgical outcomes, complication profiles, and technique refinement, reflecting the clinical priorities that have shaped ALIF practice (Stauffer and Coventry, 1972; Loguidice et al., 1988; Dennis et al., 1989; Cheung et al., 2003; Baker et al., 1993; Brau et al., 2004; Guyer et al., 2009).

A consistent signal in the most-cited ALIF corpus is the durability of foundational clinical series and radiographic observations from the era when ALIF was consolidating as a reconstructive option for painful disc disruption and pseudarthrosis in the lumbar spine. Early high-impact work emphasized measurable endpoints that remain central today, including fusion rates, segmental stability, and complications unique to anterior exposure. For example, Loguidice et al. reported fusion outcomes and complication modifiers, such as smoking status and retrograde ejaculation (Loguidice et al., 1988). These studies remain clinically relevant because they establish expectations for fusion success, complication risk, and patient selection, which continue to inform operative decision-making and perioperative counseling.

Another repeatedly cited theme is the biomechanics of disc height and the temporal behavior of distraction following ALIF with structural grafting. Dennis et al. demonstrated that postoperative distraction can diminish over time without representing failure (Dennis et al., 1989), whereas Cheung et al. described long-term disc space changes after successful autograft ALIF (Cheung et al., 2003). These findings guide the interpretation of postoperative imaging and help differentiate expected remodeling from clinically significant mechanical failure.

The citation influence of older landmark series further underscores how the field continues to treat early, carefully described experiences as reference points when newer technologies are introduced. The Mayo Clinic analysis by Stauffer and Coventry, frequently cited over decades, provides one of the best-known early institutional experiences with ALIF and the complications and indications of at that time period (Stauffer and Coventry, 1972). Even when contemporary ALIF practice has shifted toward cages, integrated fixation, image guidance, and refined patient selection, later studies still cite such early series to contextualize how risk profiles and expected benefits have changed.

The second major axis of citation impact centers on complications unique to anterior approaches, particularly vascular injury. Baker et al. highlighted the risk of major vessel injury in anterior lumbar surgery (Baker et al., 1993), and Brau et al. quantified complication rates in large cohorts (Brau et al., 2004). These studies remain highly cited because they directly influence exposure planning, multidisciplinary collaboration, informed consent, and perioperative risk management.

The results also indicate that some of the most-cited “ALIF-adjacent” work involves randomized and investigational device exemption (IDE) paradigms in which ALIF served as either the comparator or procedural component of a broader reconstructive strategy. A prominent example is the 5-year follow-up IDE trial comparing the CHARITÉ artificial disc with ALIF with BAK cages and iliac crest autograft (Guyer et al., 2009). This study is heavily cited not simply because it concerns ALIF but because it anchors long-term discussions about motion-preserving alternatives, trial endpoints, and durability of symptom improvement relative to fusion-based reconstruction. In bibliometric terms, trials of this type often achieve high citation density because they become methodological and regulatory references —informing development of subsequent device studies, payer policies, and meta-analyses—even among authors who are not primarily “ALIF specialists.”

Closely aligned with the “technical inflection point” pattern is the emergence of minimally invasive lateral or oblique corridors as influential within citation networks that overlap with the ALIF. The highly cited lateral approach interbody fusion review by Youssef et al. reflects how discourse around anterior column reconstruction expanded beyond a purely direct anterior approach into a family of access strategies designed to reduce morbidity while preserving the biomechanical advantages of interbody reconstruction (Youssef et al., 2010). From a bibliometric standpoint, this spillover is important because it suggests that when the literature is defined by anterior column objectives (disc height restoration, indirect decompression, segmental lordosis), highly cited contributions may cluster across ALIF, lateral, and oblique approaches, particularly when they provide broadly reusable conceptual frameworks (e.g., indications, complication taxonomies, and outcomes reporting conventions).

Another major citation-driving topic is the adoption of osteoinductive biologics and subsequent safety debates. The review by Carragee et al. examining adverse events and reporting practices has become a key reference in discussions of the use of biologics in spine surgery (Carragee et al., 2011). These publications illustrate how complication reporting and safety concerns can influence scholarly impact as strongly as technical innovations.

Interpreting bibliometric findings through an evidence hierarchy lens clarifies why certain randomized studies not restricted to ALIF appear as “citation attractors” in fusion-related corpora. The Swedish Lumbar Spine Study Group trial, while not an ALIF-specific paper, remains a highly cited randomized benchmark for the broader question of lumbar fusion effectiveness versus nonsurgical care in chronic low back pain populations (Fritzell et al., 2001). Such trials contribute to the “background justification” layer that many ALIF manuscripts require: even when a paper evaluates approach variants, journals and reviewers often expect authors to ground their rationale in foundational evidence that fusion can improve pain and disability in appropriately selected patients, after which approach choice becomes a next-order optimization problem. Therefore, in a bibliometric analysis, these trials function as cross-cutting “legitimizing citations” that connect ALIF-specific technique papers to the larger spine outcomes literature.

Importantly, citation frequency should not be interpreted as a proxy for methodological quality or clinical efficacy. Citation patterns reflect influence, dissemination, and historical importance, rather than intrinsic scientific validity (Bradford and Bradford, 1985; Donthu et al., 2021).

From a clinical perspective, these findings provide several practical implications for spine surgeons. First, the predominance of retrospective studies among highly cited publications highlights a relative lack of high-level evidence, underscoring the need for cautious interpretation of widely referenced data in surgical decision-making. Second, the observed shift in research focus toward complication mitigation and minimally invasive techniques reflects evolving priorities in patient safety and perioperative outcomes, which are directly relevant to contemporary surgical practice. Third, the concentration of influential evidence within specific geographic regions and institutions suggests that current practice patterns may be shaped by a limited subset of experiences, emphasizing the importance of broader validation across diverse patient populations. Collectively, these insights extend beyond traditional outcome-based reviews by identifying not only what is known, but also how the evidence base has developed and where critical gaps remain. For practicing spine surgeons, these findings emphasize that much of the evidence guiding ALIF remains based on retrospective data, reinforcing the importance of individualized surgical decision-making.

Overall, the bibliometric profile of ALIF literature reveals several important insights beyond simple citation counts. The most influential studies are predominantly retrospective clinical series, with a relative scarcity of high-level randomized evidence, highlighting a persistent gap in the evidentiary foundation of ALIF. Citation trends further demonstrate a clear temporal evolution in research priorities, shifting from early emphasis on fusion success and biomechanical stability to increasing focus on complication mitigation, implant technologies, and minimally invasive approaches. In addition, the concentration of highly cited work within a limited number of journals, institutions, and geographic regions suggests that knowledge production in ALIF remains centralized, which may influence the global generalizability of the evidence base. Collectively, these findings indicate that while ALIF is a well-established surgical technique, future research should prioritize high-quality prospective studies and broader international collaboration to address existing gaps and better inform clinical decision-making. It is also important to interpret these findings in the context of citation dynamics. Citation-based analyses inherently favor older publications, which have had more time to accumulate citations, potentially leading to underrepresentation of more recent high-quality studies. Although citation-per-year metrics partially address this limitation, they do not fully account for temporal bias. Therefore, newer studies with significant clinical relevance may not yet appear among the most highly cited works despite their potential impact on current practice.

### 4.1 Limitations

This discussion and the bibliometric results underpinning it should be interpreted in light of the methodological constraints inherent to citation-based studies. The analysis reflects the behavior of a specific indexed database at the time of extraction; therefore, coverage bias (e.g., differential indexing of older literature, non-English journals, or regional sources), citation lag, and database-specific citation counts may influence rankings and network structures. In addition, highly cited papers may be overrepresented because of “obliteration by incorporation” effects (where foundational ideas become common knowledge and are cited less) or because of non-scientific drivers of citation, such as guideline uptake, medico-legal relevance, or industry attention. Finally, bibliometric networks can identify clustering and co-occurrence but cannot establish causality or adjudicate clinical superiority among approaches. Accordingly, the findings should be interpreted as a map of scholarly influence rather than as direct evidence of treatment effectiveness or surgical superiority. Citation patterns are shaped by multiple factors beyond methodological quality, including historical precedence, academic visibility, and the influence of prominent authors and institutions. As a result, highly cited studies may gain prominence through dissemination dynamics and network effects rather than definitive clinical superiority.

In addition, the observed clustering of publications and citations at the country and institutional level may reflect the influence of established research groups and regionally dominant authors. In surgical practice, such dynamics may contribute to the preferential adoption and reinforcement of specific techniques within particular geographic or academic settings. Consequently, certain studies may become widely cited and function as de facto reference standards within these networks, even in the absence of definitive evidence demonstrating superiority over alternative approaches. This underscores the importance of critically appraising even highly cited literature and highlights the potential divergence between citation impact and evidence-based validity in surgical decision-making.

### 4.2 Strengths

A principal strength of this study is that it systematically synthesizes the highly influential ALIF literature into an interpretable structure that clinicians, researchers, and trainees can use to understand how the field developed and where the influence concentrates. By combining descriptive indicators (production and citation trajectories), source concentration, author and institutional prominence, and collaboration mapping, this study offers a multidimensional view that is difficult to obtain from a narrative review alone. This approach also improves transparency and reproducibility by using established bibliometric tools and clearly defined outputs, enabling future investigators to replicate the workflow, update it with newer time windows, or apply comparable methods to related procedures (e.g., OLIF, LLIF, TLIF) for comparative intellectual mapping.

### 4.3 Future perspectives

Future work can extend these findings by moving from “who and what is most cited” to “how influence relates to evidence quality and patient-centered outcomes.” One possible strategy is to link citation strata (e.g., top-decile cited papers vs. mid-tail) with study design features, risk-of-bias markers, and outcome domains such as function, return to work, reoperation, adjacent segment disease, and approach-specific complications, thereby testing whether citation prominence tracks with evidentiary robustness. Another important direction is field-normalized and time-normalized analyses that reduce bias toward older publications and allow contemporary high-impact work to be assessed. Finally, integrating altmetrics, open science indicators, and registry-linked trial evidence could provide a more complete picture of translational impact, capturing not only academic influence but also practice change, policy consequences, and patient-facing dissemination.

## 5 Conclusion

This bibliometric analysis provides a comprehensive overview of the most influential literature on ALIF, highlighting the field's intellectual structure, thematic priorities, and collaborative networks. The findings demonstrate that highly cited ALIF research predominantly focuses on surgical outcomes, complication profiles, and technical refinements, reflecting clinical applicability as a major driver of the scholarly impact. Key contributions originate from a concentrated group of journals, authors, and institutions, with the United States leading the global citation influence. While foundational clinical series and radiographic studies continue to serve as essential reference points, emerging innovations such as osteobiologics and minimally invasive approaches also shape the evolving discourse. Importantly, citation frequency reflects scholarly influence rather than intrinsic evidence quality; therefore, clinical decisions should remain grounded in critical appraisal of study design, outcomes, and complication data. The study's systematic approach provides a structured overview of influential publications that may assist clinicians and researchers in contextualizing the ALIF evidence base and understanding its developmental trajectory.

## Declaration of interests

The authors declare that they have no known competing financial interests or personal relationships that could have appeared to influence the work reported in this paper. No external funding, grants, or industry support was received for the conduction of the study, analysis, or preparation of the manuscript. The authors have not received honoraria, consulting fees, stocks, or other forms of compensation from entities with an interest in the subject matter discussed. All authors had full access to the data and take responsibility for the integrity and accuracy of the analysis. The manuscript has not been published previously and is not under consideration elsewhere.
